# Television Viewing and Snacking Behaviors of Fourth- and Eighth-Grade Schoolchildren in Texas

**Published:** 2009-06-15

**Authors:** Deanna M. Hoelscher, Amanda M. Vader, Scott T. Walters, T. Robert Harris

**Affiliations:** Michael and Susan Dell Center for Advancement of Healthy Living, University of Texas School of Public Health, Austin Regional Campus; affiliation; affiliation; University of Texas School of Public Health, Dallas, Texas

## Abstract

**Introduction:**

Child and adolescent overweight is a serious health issue. Both snacking and television watching have been associated with childhood overweight, but the relationships have not been well examined in a multiethnic population. The aim of this study was to examine relationships between weekday television viewing, snack consumption, consumption of foods advertised on television, and overweight status of a multiethnic sample of fourth- and eighth-grade schoolchildren in Texas.

**Methods:**

This study is a secondary analysis of data from the School Physical Activity and Nutrition monitoring system, a validated survey with objective measures of height and weight. The sample of 11,594 children in the fourth and eighth grades was weighted to provide data representative of children in Texas public schools. Children were categorized on the basis of self-reported daily television viewing, snack consumption, and consumption of foods advertised on television. Multiple logistic regression was used to analyze, by grade level, the differences in the prevalence of overweight by category.

**Results:**

Television viewing, frequency of snack consumption, and consumption of foods advertised on television were all positively related to one another. In general, both consuming more snacks and foods advertised on television were associated with reduced odds of overweight regardless of the amount of television watched.

**Conclusion:**

The results suggest that the relationships between weekday snacking behaviors and television viewing in a multiethnic population are complicated. When these behaviors are clustered, overweight status may be related more to the number of snacks consumed than to the amount of television watched. To determine the exact relationship, additional research, especially among Hispanic children, is warranted.

## Introduction

Children in the United States today face what is widely viewed as an epidemic of overweight. Prevalence increased markedly in the past decades and has remained high in recent years (1,2). Among the many speculated causes of childhood overweight are dietary and television-viewing behaviors, which each play a complicated role. Snacking, a common dietary behavior among US children, can lead to consumption of excess calories and result in overweight. Although evidence from cross-sectional and longitudinal studies supports a relationship between snacking and overweight among children (3,4), 2 prospective longitudinal studies found no relationship (5,6). Increases in sedentary activities such as watching television, playing video games, and surfing the Internet are also often cited as causes of childhood overweight. Several longitudinal and cross-sectional studies have analyzed the relationship between television viewing and overweight; many have found a positive association (7,8). A meta-analysis found a small effect size between television viewing and body fatness in children (9). The relationship was significant, but the authors concluded that overweight is unlikely to be explained by television viewing alone.

As a sedentary activity, watching television can decrease a child's energy expenditure because it may replace more physically active pursuits (10). It may also have a role in increasing energy consumption because television viewing and snacking often occur together. A longitudinal study of young girls (aged 5 years at baseline) showed a positive correlation between television viewing and eating snacks while watching television (*r* = 0.33 for girls from overweight families and *r* = 0.29 for girls from families who were not overweight) (4). Other studies have positively associated television viewing with consumption of energy-dense snacks (5), sweet snacks, and high-energy drinks (11), as well as overall energy intake (12).

Not only do children often snack while watching television but their food choices also appear to be influenced by the television commercials and programming (13). The Kaiser Family Foundation (14) reported that half of all commercial airtime on children's television shows is for food, mostly foods that are high in fat, oil, or sugar (15,16). The effects of advertising to children can be seen at the ecological level. Data from 10 countries found a positive association between the prevalence of overweight in children and the number of children's television advertisements for energy-dense foods (17).

Although many studies have examined the association of television watching and snacking behaviors among children, few have been conducted in multiethnic populations that include a substantial number of Hispanic children. The objective of this study was to examine the relationships between television viewing, snacking behaviors, and overweight among a multiethnic and representative sample of Texas children in elementary and middle schools.

## Methods

This study used data from fourth- and eighth-grade students participating in the School Physical Activity and Nutrition (SPAN) monitoring system during the 2000-2001 and 2001-2002 school years (18). SPAN was developed as a surveillance system for body mass index (BMI) changes in a representative sample of Texas schoolchildren. One grade level was targeted to represent each developmental group: 4th grade for elementary school, 8th grade for middle school, and 11th grade for high school. The sampling plan and frame have been described previously (18) and consisted of 439 school districts, accounting for 91.5% of the Texas public school population enrolled in the 4th, 8th, and 11th grades. (Data for 11th-grade students are not discussed in this article.)

SPAN was approved by the institutional review board at the University of Texas Health Science Center at Houston, the Texas Department of Health institutional review board, and participating school districts. Informed consent was provided by parents or guardians, and children were required to assent before any measurement occurred.

### Data collection

Students' height and weight were measured by using a standard protocol (18). BMI was calculated from the measured height and weight as the weight in kilograms divided by height in meters squared. For children, the Centers for Disease Control and Prevention (CDC) defines obesity as a BMI greater than or equal to the 95th percentile for age and sex, overweight as a BMI greater than or equal to the 85th percentile but less than the 95th percentile, and normal as a BMI less than the 85th percentile but greater than the 5th percentile (www.cdc.gov/growthcharts/).

Self-reported demographic information; nutrition behaviors, attitudes, and knowledge; and physical activity behaviors were assessed by the SPAN questionnaire. The questionnaires were administered on Tuesday through Friday so that responses to questions concerning the previous day reflected weekday behavior. The questionnaire was found to have acceptable reliability and validity compared with similar dietary assessment instruments for both elementary- and secondary-level students (19-21).

Daily television watching, snacking behavior, and physical activity (used as a covariate) were determined by single questions. The daily consumption of the foods most frequently advertised on television shows targeted to children (television foods) was assessed by 5 questions measuring punch, sports drinks, and other fruit-flavored drinks; sodas and soft drinks; frozen desserts; sweet rolls, doughnuts, cookies, brownies, pies, and cakes; and chocolate candy (15). A composite score for the frequency of television foods was calculated by summing the responses to these 5 questions. The questions and possible responses are listed in the Appendix.

### Data analyses

We used Stata 9.0 (StataCorp, College Station, Texas) to analyze all data. The Stata survey procedure was used to account for the stratified, weighted, cluster-sampling procedure when conducting hypotheses testing and creating confidence intervals (CIs).

Weighted frequencies of television viewing, snack consumption, and foods advertised on television were calculated for each grade and sex. For each grade, we calculated odds ratios (ORs) with 95% CIs to demonstrate whether overweight status occurs more frequently with high levels of each of 3 independent variables (television viewing, snack consumption, or television foods). ORs were adjusted for sex, race and ethnicity, and physical activity.

To examine the relationships between television viewing, snack consumption, and television foods, we dichotomized the 3 variables as follows: daily television viewing, 0-2 hours of television vs 3 or more hours of television; daily snack consumption, 0-1 snacks vs 2 or more snacks; and daily television foods,  0-3 television foods vs 4 or more television foods.

The differences in the prevalence of overweight based on television viewing and snack consumption were analyzed by using a multiple logistic regression strategy. First, we used a typology of the television viewing and snack consumption variables to classify each child as follows: 1) 0-2 hours of television per day and 0-1 snack per day (low television/low snacks); 2) 0-2 hours of television per day and 2 or more snacks per day (low television/high snacks); 3) 3 or more hours of television per day and 0-1 snack per day (high television/low snacks); or 4) 3 or more hours of television per day and 2 or more snacks per day (high television/high snacks). We performed a separate regression analysis for each grade by using sex, race and ethnicity, and physical activity as covariates. We calculated adjusted ORs to compare the prevalence of overweight in 3 of the categories (low television/high snacks, high television/low snacks, and high television/high snacks) to the prevalence of overweight in low television/low snacks category. We used a similar multiple logistic regression strategy to analyze the differences in the prevalence of overweight based on television viewing and television foods.

## Results

The unweighted sample consisted of 6,235 fourth-grade students and 5,359 eighth-grade students. The weighted distributions of age, sex, race and ethnicity, and BMI category among the SPAN participants are shown by grade (Table 1).

The frequencies of television viewing, snack consumption, and television foods are presented by grade and sex (Table 2). All 3 behaviors were common among SPAN participants, and the distribution was similar between boys and girls in the same grade. The general trend between grades was that more fourth-grade students reported low frequencies, whereas more eighth-grade students reported high frequencies of all 3 behaviors. For example, only 1.8% of eighth-grade girls and 1.2% of eighth-grade boys reported no daily television watching, whereas 21.5% of fourth-grade girls and 15.5% of fourth-grade boys did not watch any television (Table 2).

### Relationship between television viewing and snacking behaviors

Fourth-grade students who ate 2 or more snacks per day were 77% more likely to watch 3 or more hours of television per day than were those who ate 1 or fewer snacks per day. Eighth-grade students who reported eating 2 or more snacks per day were 44% more likely to watch 3 or more hours of television per day than those who ate no snacks or 1 snack. Students who ate 4 or more television foods per day were more likely to watch 3 or more hours of television per day than those who ate 3 or fewer television foods per day. Furthermore, both fourth- and eighth-grade students who ate 2 or more snacks per day were more likely than those who ate 1 or fewer snacks per day to also eat 4 or more television foods per day.

### Prevalence of overweight by television viewing and snacking behaviors

Eighth-grade students who watched television 1 to 2 hours per day were more likely to be overweight than were those who watched less than 1 hour per day (Table 3). We found no significant relationships between television viewing and overweight for fourth-grade students. Children in both grades who ate 2 snacks per day and 3 or more snacks per day were less likely to be overweight than were those who ate none. Fourth-grade students who ate 1 snack per day were 44% less likely to be overweight than were those who ate no snacks. Eighth-grade students who ate 2 or 3, and 4 or more television foods per day were less likely to be overweight than those who ate no television foods or 1 television food per day. We saw no difference in the likelihood of overweight by television foods for fourth-grade students (Table 3).

Fourth-grade students who watched little television and ate many snacks were 45% less likely to be overweight than were those who watched little television and ate few snacks (Table 4). Eighth-grade students who watched little television and ate many snacks per day and those who watched many hours of television and ate many snacks per day were also less likely to be overweight than were those who watched little television and ate few snacks.

Eighth-grade students who watched little television and ate many television foods per day and eighth-grade students who watched many hours of television and ate many television foods per day were less likely to be overweight than were members of the referent group (Table 4).

## Discussion

In a multiethnic student population in Texas, snack frequency and television foods were positively associated with television viewing. In general, watching more television was associated with being overweight, although this relationship was significant only among eighth-grade students who watched 1 to 2 hours of television per day. In contrast, eating snacks and television foods was less likely to be associated with being overweight. In addition, eating several snacks and television foods per day was associated with a lower chance of being overweight regardless of the amount of television watched, and the relationship was more evident among eighth-grade students.

Rates of television watching among SPAN participants were similar to rates reported in other studies (10,12). As in some previous studies (7,8), we found a positive association between television viewing and overweight among children, whereas others have found little or no association (9,10). These mixed results may be related to the different measures of daily television viewing (weekday, weekend, or both). SPAN collected weekday data only, and weekend television watching was not measured, possibly influencing the results. The frequency of snacking among SPAN participants was similar to that published by other researchers, and most participants reported consuming at least 1 snack per day (22,23). As in a previous study (6), we found that snacking was associated with a lower risk of overweight. Snacking itself is not an unhealthy habit; the American Dietetic Association recommends that children eat 2 to 3 healthy snacks a day (24). SPAN participants who ate many snacks each day may have been eating small portions or healthy snacks, or eating less at meal time. Knowledge of type of snack foods, serving sizes of snacks, and total daily calorie intake would provide further information about the role of snacks in overweight.

SPAN participants reported eating television foods at rates similar to those reported in previous studies (12). The television foods variable differs from the snack variable in that the type of food is specified. A snack can be "healthy" or "unhealthy," whereas the television foods variable includes mostly unhealthy items. Surprisingly, the trend among children in Texas was that increased consumption of the unhealthy television foods was associated with a lower risk of overweight, although this relationship was significant only for eighth-grade students who ate 2 or 3 and 4 or more television foods per day.

### Relationship between television viewing and snacking behaviors

Television viewing and snack consumption were positively associated for students in the fourth and eighth grades, which supports the results of previous studies on the relationship between television viewing and snacking (4,5,11). Additionally, the relationship between television viewing and television foods was straightforward; the more television foods eaten, the more television watched. Similar results have been found by many researchers and attest to the power of food advertisements on television (12,13,17,25-27). Although SPAN had questions on television viewing and snacking behaviors, there was no question linking snacking behaviors to watching television; thus, we cannot know if the snacks or television foods were eaten while watching television or eaten directly because of commercials.

### Prevalence of overweight by television viewing and snacking behaviors

Generally, children who reported eating more snacks per day were less likely to be overweight than those who consumed fewer snacks per day regardless of hours of television viewing. The pattern for television viewing and television foods was similar. These results were surprising because of the unhealthy nature of the television foods. Although these analyses were adjusted for sex, race and ethnicity, and physical activity, they were not adjusted for other factors that might be associated with the development of overweight, such as socioeconomic status, other sedentary activities, total caloric intake, or overall quality of diet. The SPAN survey was designed to examine weekday habits; including weekend and weekday habits may yield different results, particularly because the number of food advertisements aimed at children is high on weekend days, especially Saturday morning (28). Although our analyses were adjusted for race and ethnicity, the results of this study may have been influenced by the large proportion of Hispanic SPAN participants, in a way that other, less multiethnic studies are not. Finally, the SPAN survey had a truncated number of options for each of the behaviors (eg, 3 or more snacks per day). A different relationship might exist if we had been able to distinguish between higher frequencies (5 snacks) and those in the middle (3 snacks).

### Strengths and limitations

The major strength of this study is that it was based on a large, diverse sample that is representative of fourth- and eighth-grade children in Texas public schools. The SPAN questionnaires were tested for reliability and validity before being used (19-21), and BMI was calculated by using objectively measured height and weight.

This study is limited by the use of cross-sectional data and self-reported data for television viewing, snacking behaviors, and race/ethnicity. Because the study was cross-sectional, we were able to look only at relationships that existed when the questionnaire was administered. Children self-reported their television viewing, snacking behaviors, and ethnicity, which has the potential to create inaccuracies or skew the data. Overweight children are more likely than children of normal weight to underreport their food intake (29,30). A selective underreporting of food or dieting by restricting certain foods, such snacks and television foods, by overweight children in the SPAN study could have contributed to the results. Additionally, SPAN assessed the frequency of consumption of snacks, not portion sizes, and hours of television watched, not type of programming (eg, movies, educational, or commercial television), which limits our ability to interpret the meaning of eating 2 snacks or watching 2 hours of television without having more information about either behavior.

### Conclusion

Snacking, eating foods commonly advertised on television, and watching television were all frequently reported by students participating in the SPAN survey. The results suggest that the relationships between weekday snacking behaviors and television viewing in a multiethnic population are complicated, and relationships to overweight status may be related more to the number of snacks than to the amount of television watched. To determine the exact relationship, additional research into the food type and serving size of snacks, timing of snack consumption with television watching, the amount and type of television watched, and racial/ethnic differences in snacking and television viewing, especially among Hispanic children, is warranted.

## Figures and Tables

**Table 1:** Demographic Characteristics of Texas Schoolchildren, School Physical Activity and Nutrition Survey, 2000-2001 and 2001-2002 School Years

Characteristic	Fourth Grade (n = 6,235)^a^	Eighth Grade (n = 5,359)^b^
**Age, mean (SD), y**	9.7 (0.6)	13.7 (0.6)
**Sex, %**		
Girls	48.1	45.4
Boys	51.9	54.6
**Race/ethnicity, %**		
African American	11.8	11.2
Hispanic	45.3	40.9
White/other^c^	42.9	47.9
**Body mass index (BMI) category, %**		
Normal^d^	56.2	63.5
At risk of overweight^e^	18.2	17.8
Overweight^f^	25.6	18.7

Abbreviations: CDC, Centers for Disease Control and Prevention.

a Represents unweighted fourth-grade sample. The results presented in the table are based on the weighted sample (fourth grade, n = 309,863).

b Represents unweighted eighth-grade sample. The results presented in the table are based on the weighted sample (eighth grade, n = 288,539).

c White/other category consists of non-Hispanic white, Asian, Pacific Islander, Native American, and other.

d BMI <85th percentile of BMI for age and sex from CDC growth charts.

e BMI ≥85th percentile but <95th percentile for age and sex from CDC growth charts.

f BMI ≥95th percentile for age and sex from CDC growth charts.

**Table 2:** Television Viewing, Snack Consumption, and Consumption of Foods Advertised on Television Among Texas Schoolchildren, by Grade and Sex, School Physical Activity and Nutrition Survey, 2000-2001 and 2001-2002 School Years

	Four**th Grade % (95% CI)**		Eigh**th Grade % (95% CI)**	

	**Girls**	**Boys**	**Girls**	**Boys**
**Television viewing^a^, no. of hours per day**				
0	21.5 (18.0-25.3)	15.5 (12.9-18.4)	NA	NA
1	35.1 (32.1-38.3)	34.2 (30.4-38.2)	NA	NA
2	22.2 (18.2-26.7)	22.8 (19.5-26.4)	NA	NA
3 or more	21.3 (18.1-24.8)	27.6 (24.0-31.6)	NA	NA
0	NA	NA	1.8 (1.2-2.8)	1.2 (0.7-2.2)
<1	NA	NA	16.0 (13.2-19.3)	12.8 (9.9-16.4)
1 or 2	NA	NA	33.8 (29.7-38.0)	35.9 (32.0-39.9)
3 or 4	NA	NA	26.7 (23.7-29.9)	27.1 (21.8-33.2)
>4	NA	NA	21.8 (18.1-25.9)	23.0 (19.9-26.4)
**Snack consumption, no. of snacks per day**				
0	20.3 (17.2-24.1)	25.9 (22.0-30.2)	16.9 (12.9-21.9)	17.9 (12.9-24.4)
1	56.6 (52.7-60.5)	48.7 (45.1-52.3)	39.2 (34.3-44.4)	34.9 (30.1-40.1)
2	16.9 (13.8-20.5)	16.2 (12.9-20.1)	28.5 (24.3-33.2)	26.9 (21.7-32.8)
>3	6.2 (4.2-8.9)	9.3 (6.9-12.3)	15.3 (13.1-17.8)	20.3 (17.0-23.9)
**Foods advertised on television, no. of foods per day**				
0	7.6 (5.4-10.5)	9.9 (8.0-12.3)	4.6 (3.3-6.4)	4.7 (3.3-6.6)
1	17.5 (14.7-20.7)	15.8 (12.1-20.4)	14.0 (10.8-18.0)	11.1 (8.4-14.7)
2 or 3	42.3 (36.5-48.3)	40.1 (35.7-44.6)	37.5 (32.1-43.2)	34.6 (30.5-39.0)
>4	32.6 (27.9-37.7)	34.1 (29.7-38.8)	43.9 (35.5-52.6)	49.6 (44.2-55.0)

Abbreviation: NA, not applicable.

a The 2 sets of possible responses differed for fourth-grade students and eighth-grade students.

**Table 3:** Overweight by Television Viewing, Snack Consumption, and Consumption of Foods Advertised on Television, School Physical Activity and Nutrition Survey, 2000-2001 and 2001-2002 School Years^a^

	Fourth Grade OR (95% CI)	Eighth Grade OR (95% CI)
**Television viewing, hours per day**		
0	1.00 [reference]	NA
1	1.43 (0.93-2.19)	NA
2	1.33 (0.73-2.43)	NA
3	1.32 (0.80-2.17)	NA
0 or <1	NA	1.00 [reference]
1 or 2	NA	1.41 (1.03-1.92)
3, ≥4	NA	0.89 (0.51-1.57)
>4	NA	1.35 (0.97-1.88)
**Snack consumption, no. snacks per day**		
0	1.00 [reference]	1.00 [reference]
1	0.56 (0.40-0.78)	0.87 (0.57-1.32)
2	0.49 (0.34-0.70)	0.49 (0.26-0.90)
≥3 or more	0.34 (0.18-0.62)	0.39 (0.23-0.67)
**Foods advertised on television, no. foods per day**		
0 or 1	1.00 [reference]	1.00 [reference]
2 or 3	0.88 (0.57-1.35)	0.60(0.38-0.94)
≥4	0.71 (0.45-1.12)	0.37 (0.25-0.54)

OR, odds ratio; CI, confidence interval; NA, not applicable.

a Odds ratios were adjusted for sex, race and ethnicity, and physical activity. Overweight is defined as a body mass index greater than or equal to the 95th percentile for age and sex.

**Table 4:** Overweight by Television Viewing, Snack Consumption, and Consumption of Foods Advertised on Television^a^

Variables	Fourth Grade OR (95% CI)	Eighth Grade OR (95% CI)
**Overweight by television viewing and snack consumption**		
Low television/low snacks^b^	1.00 [reference]	1.00 [reference]
Low television/high snacks^c^	0.55 (0.40-0.75)	0.41 (0.25-0.68)
High television/low snacks^d^	0.91 (0.67-1.25)	0.78 (0.55-1.09)
High television/high snacks^e^	0.86 (0.50-1.46)	0.46 (0.31-0.69)
**Overweight by television viewing and consumption of foods advertised on television**		
Low television/low television foods^f^	1.00 [reference]	1.00 [reference]
Low television /high television foods^g^	0.69 (0.47-1.03)	0.39 (0.25-0.61)
High television/low television foods^h^	0.95 (0.67-1.34)	0.79 (0.54-1.17)
High television/high television foods^i^	0.88 (0.55-1.40)	0.53 (0.39-0.72)

OR, odds ratio; CI, confidence interval.

a Odds ratios were adjusted for sex, race and ethnicity, and physical activity. Overweight is defined as a body mass index greater than or equal to the 95th percentile for age and sex.

b ≤2 hours of television per day and 0 to 1 snack per day.

c ≤2 hours of television per day and ≥2 snacks per day.

d ≥3 or more hours of television per day and 0 to 1 snack per day.

e ≥3 hours of television per day and ≥2 snacks per day.

f ≤2 hours of television per day and 0 to 3 television foods per day.

g ≤2 hours of television per day and ≥4 television foods per day.

h ≥3 hours of television per day and 0 to 3 television foods per day.

i ≥3 hours of television per day and ≥4 television foods per day.

## Appendix. Relevant Questions and Possible Responses on the Elementary and Secondary School SPAN Questionnaires

**Table:**

Elementary School Questionnaire		Secondary School Questionnaire	

Question	Possible Answers	Question	Possible Answers
Yesterday, how many hours did you watch television or video movies?	I didn't watch television yesterday 1 hour 2 hours 3 hours or more	How many hours per day do you usually watch television or video movies?	I don't watch television or video movies Less than 1 hour a day 1-2 hours a day 3-4 hours a day More than 4 hours a day
Yesterday, did you have a snack? A snack is food or drink that you eat or drink before, after, or between meals.	No, I didn't have any snacks yesterday. Yes, I had a snack 1 time yesterday. Yes, I had a snack 2 times yesterday. Yes, I had a snack 3 or more times yesterday.^a^	Yesterday, how many times did you eat or drink a snack? A snack is any food or beverage that you eat or drink before, after, or between meals.	None 1 time 2 times 3 or more times^a^
Yesterday, did you do any exercise that made your heart beat fast and made you breathe hard for at least 20 minutes? (For example: basketball, running or jogging, fast dancing, swimming laps, tennis, fast bicycling, or similar aerobic activities.)	Yes No	On how many of the past 7 days did you exercise or take part in physical activity that made your heart beat fast and made you breathe hard for at least 20 minutes? (For example: basketball, soccer, running or jogging, fast dancing, swimming laps, tennis, fast bicycling, or similar aerobic activities.)	0 days 1 day 2 days 3 days 4 days 5 days 6 days 7 days

a Questions for television food items (punch, sports drinks and other fruit-flavored drinks; sodas and soft drinks; frozen desserts; sweet rolls, doughnuts, cookies, brownies, pies and cakes; and chocolate candy) had the same response options as the snack question.
